# iTRAQ-based proteomic analysis of plasma reveals abnormalities in lipid metabolism proteins in chronic kidney disease-related atherosclerosis

**DOI:** 10.1038/srep32511

**Published:** 2016-09-07

**Authors:** Magdalena Luczak, Dorota Formanowicz, Łukasz Marczak, Joanna Suszyńska-Zajczyk, Elżbieta Pawliczak, Maria Wanic-Kossowska, Maciej Stobiecki

**Affiliations:** 1 European Centre for Bioinformatics and Genomics, Institute of Bioorganic Chemistry, Poznan, 61-138, Poland; 2 Institute of Chemical Technology and Engineering, Poznan University of Technology, Poznan, 60-965, Poland; 3 Department of Clinical Biochemistry and Laboratory Medicine, Poznan University of Medical Sciences, Poznan, 60-780, Poland; 4 Department of Biochemistry and Biotechnology, Poznan University of Life Sciences, Poznan, 60-632, Poland; 5 Department of Nephrology, Transplantology and Internal Medicine, Poznan University of Medical Sciences, Poznan, 60-355, Poland

## Abstract

Patients with chronic kidney disease (CKD) have a considerably higher risk of death due to cardiovascular causes. Using an iTRAQ MS/MS approach, we investigated the alterations in plasma protein accumulation in patients with CKD and classical cardiovascular disease (CVD) without CKD. The proteomic analysis led to the identification of 130 differentially expressed proteins among CVD and CKD patients and healthy volunteers. Bioinformatics analysis revealed that 29 differentially expressed proteins were involved in lipid metabolism and atherosclerosis, 20 of which were apolipoproteins and constituents of high-density lipoprotein (HDL) and low-density lipoprotein (LDL). Although dyslipidemia is common in CKD patients, we found that significant changes in apolipoproteins were not strictly associated with changes in plasma lipid levels. A lack of correlation between apoB and LDL concentration and an inverse relationship of some proteins with the HDL level were revealed. An increased level of apolipoprotein AIV, adiponectin, or apolipoprotein C, despite their anti-atherogenic properties, was not associated with a decrease in cardiovascular event risk in CKD patients. The presence of the distinctive pattern of apolipoproteins demonstrated in this study may suggest that lipid abnormalities in CKD are characterized by more qualitative abnormalities and may be related to HDL function rather than HDL deficiency.

Chronic kidney disease (CKD) is a progressive loss of renal function lasting at least 3 months and characterized by a decreased glomerular filtration rate (GFR) and proteinuria, as diagnosed on the basis of the urinary albumin: creatinine ratio^1^,^2^. Patients with CKD have an absolute increased risk for cardiovascular disease (CVD), which is inversely related to GFR. Cardiovascular mortality is approximately three-fold higher in patients with stage 4 CKD than in individuals with normal kidney function^3^. Moreover, the risk of death due to CVD is greater than the risk of requiring renal replacement therapy^1^. This risk increases dramatically (10-30-fold higher) in patients with end-stage renal disease (ESRD) when they start dialysis treatment^4^. In the general population, there are strong associations between cholesterol fractions and the risk of atherosclerosis. In classical CVD, a high concentration of total cholesterol and a high concentration of the atherogenic fraction of low-density lipoprotein (LDL) particles, as well as a low concentration of anti-atherogenic high-density lipoprotein (HDL) particles, are associated with the prevalence of cardiovascular events. Normal HDL function is characterized by reverse cholesterol transport from peripheral cells to the liver. HDL protects LDL against oxidation and suppresses systemic inflammation^5^. Therefore, HDL deficiency is key in perpetuating chronic inflammation and oxidative stress, thus leading to atherosclerosis. Epidemiological data have shown that in CKD, the link between cholesterol and lipoprotein fractions is not as straightforward as that in the general population. CKD is frequently accompanied by reduced plasma HDL concentrations and normal or even low serum total cholesterol and LDL concentrations^6^,^7^. In CKD patients, higher total cholesterol and LDL values may even be associated with greater survival and a lower risk of CVD^8^,^9^. Moreover, lowering LDL cholesterol with statin therapy is effective in reducing the risk of cardiovascular morbidity and mortality only among people with mild degrees of renal impairment but not in patients at the most advanced CKD stages, i.e., ESRD^10^. The association between low serum cholesterol concentrations and higher mortality, especially in ESRD patients, is probably related to systemic inflammation or malnutrition, both of which have a cholesterol-lowering effect^11^. Therefore, the question of whether atherosclerosis in patients with CKD is a different process than that in patients with classical CVD persists. Given the association between CVD and CKD, only a direct analysis of the proteomes of both conditions in one study may provide insight into the answer. This article focuses on the alterations in abundance of proteins involved in lipid transport, metabolism and atherosclerotic plaque formation in patients with different stage of CKD as well as patients with “classical” CVD.

## Results

### Sample quality control and data processing

**Figure 1:**
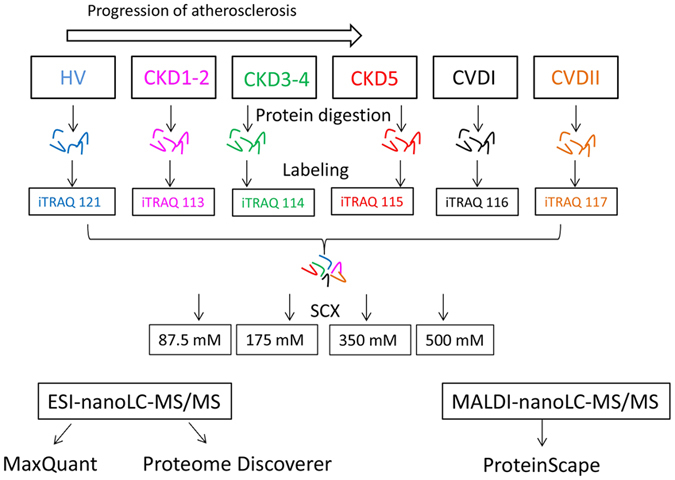
Workflow of the experimental strategy used in this study. Plasma samples from six experimental groups were trypsin digested, labeled with isobaric tags, pooled and then purified and fractionated using SCX method. Quantitative proteomic analyses were simultaneously performed using ESI-nanoLC-MS/MS and MALDI-nanoLC-MS/MS and then obtained data were analyzed with three types of software: MaxQuant, ProteinScape and Proteome Discoverer. Only proteins identified by all software were found to have a differential accumulation level.

**Figure 2:**
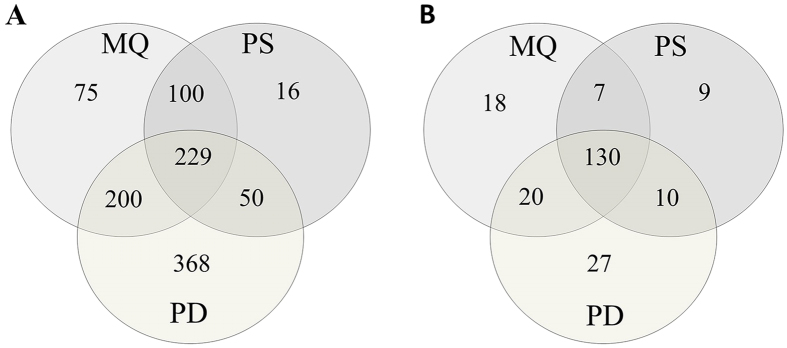
A Venn diagram comparing the results from the MaxQuant (MQ), Proteome Discoverer (PD), and ProteinScape (PS) software in ten iTRAQ experiments. (**A**) A total of 1,038 unique proteins were identified, 229 of which were common between the approaches. (**B**) A total of 221 differentially expressed proteins were identified in all the experiments, 130 of which were common between the approaches.

The plasma samples from patients with different stages of CKD, patients with CVD, and healthy volunteers (HVs) were studied using iTRAQ labeling and off-line and on-line nanoLC-MS/MS. We compared results from iTRAQ analyses using two different platforms: ESI-nanoLC-MS/MS and MALDI-nanoLC-MS/MS. Then, the obtained data were analyzed using three software solutions: MaxQuant (MQ), ProteinScape (PS), and Proteome Discoverer (PD). The workflow of the study is presented on Fig. 1. As a result, 604, 395, and 847 proteins were identified with a 1% false discovery rate (FDR) and a minimum of 2 peptides using MQ, PS, and PD software, respectively (Fig. 2A). A total of 1,038 unique proteins were identified with a minimum of 2 peptides. For all identified in MQ and PS proteins, only 12.4% and 4% were unique for both software. However, up to 43.4% proteins were identified only by PD. On the other hand, PD analysis showed that the percentage overlap between the triplicate injections was above 84% at the protein level. The percentage overlap between the biological replicates from the same experimental group was 70% at the protein level.

**Figure 3:**
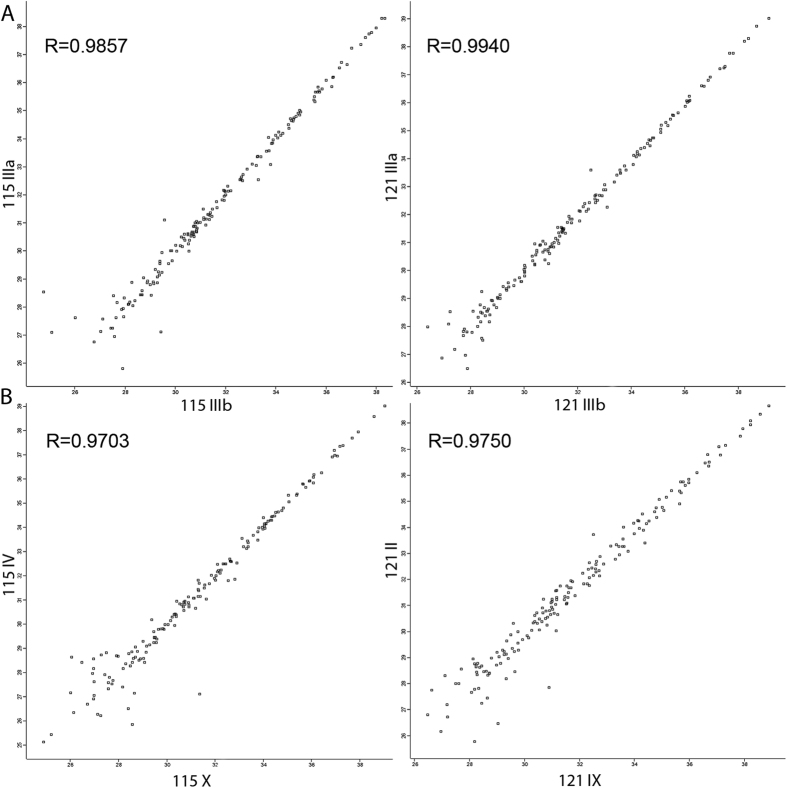
Representative correlation plots of 115 and 121 reporter ion intensities from two technical (A) and two biological (B) experiments. The Pearson correlation coefficient is provided for each plot.

The reproducibility of technical and biological replicates was assessed by scatter plotting and correlation coefficient determination based on reporter ion intensity parameters. Exemplary scatter plots comparing the 115 and 121 reporter ion intensities obtained during ESI-MS/MS experiments (Fig. 3) showed very good correlation, with r = 0.98 (for the 115 reporter ion) and 0.99 (for the 121 reporter ion) for the technical replicates and r = 0.97 for the biological replicates. The correlation analysis of reporter ion signals between technical and biological replications calculated for other reporter ions revealed Pearson coefficients between 0.79 and 0.99.

### Quantitative analysis of plasma proteins

All obtained data sets were then statistically analyzed to find differentially expressed proteins. Quantitative analysis led to the identification of 177 (MQ), 158 (PS), and 189 (PD) proteins with a minimum of 2 labeled peptides, with variability in the reporter ion signal below 30%, a threshold greater than 1.5, and P values below 0.05 (ANOVA), which differentiated the analyzed groups of patient. For all identified by MQ 177 differential proteins only 18 were unique for MQ what stands for about 10.2% of identified proteins. Similar results were obtained for other software: 14.3% and 5.7% unique proteins were identified for PD and PS, respectively. Only 130 proteins identified by all software were considered as differentiating proteins (Fig. 2B); these proteins are presented in Supplementary Material 1A.

**Figure 4:**
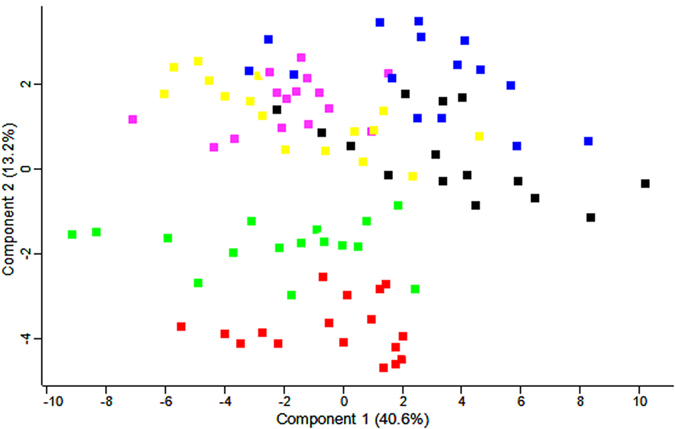
PCA of the reporter ion intensities obtained from the plasma of HVs (blue), CKD1-2 (yellow), CKD3-4 (green), CKD5 (red), CVDI (black) and CVDII (pink) patients. Calculations were performed with Perseus.

Comparative proteomic analyses were performed between HVs and each of the CKD or CVD groups and between neighboring groups of CKD or CVD patients. Analysis of the differentially expressed proteins revealed that 46 proteins distinguished the HV and CKD1-2 groups and that 63 and 78 proteins distinguished the HV and CKD3-4 groups and HV and CKD5 groups, respectively. The comparison between HV and CVDI/CVDII patients revealed 40 and 44 differentially expressed proteins. Only 9 proteins differentiated the CVDI and CVDII patients. PCA differentiated all analyzed experiment group, it is especially clear for CKD3-4 and CKD5 patients (Fig. 4).

### Functional and disease annotations of differential proteins

We used an analysis tool, DAVID, to detect enriched GO annotations in the 130 differentially expressed proteins. The data were classified based on the diseases to which they contribute. The main enriched DAVID categories in terms of disease were cardiovascular disease (with a Benjamini corrected P value 1.7e-9), including 46% of the differentially expressed proteins, and atherosclerosis (P = 1.9e-8), including 23.3% of the proteins with altered accumulation. Both categories contained 20 apolipoproteins and other proteins that participate in lipid metabolism and atherosclerotic plaque formation. Among the DAVID disease categories, myocardial infarction and coronary heart disease were also enriched (P = 2.4e-8 and 1.1e-7).

The results from the bioinformatics analysis revealed that 29 differentially expressed proteins were involved in lipid metabolism and atherosclerotic plaque formation; these proteins included diverse classes of apolipoproteins, angiogenin, angiotensinogen, adiponectin, cholinesterase, fibrinogen, haptoglobin, antigen CD14, and serum amyloid proteins (Table 1). The relative abundance and fold changes of these proteins are presented in Supplementary Material 1A. Because proteins involved in lipid metabolism and atherosclerotic plaque formation constituted up to 22% of the identified differentially expressed proteins, in the next analyses we focus only on this set of molecules.

### Differentially expressed proteins associated with lipid metabolism

**Figure 5:**
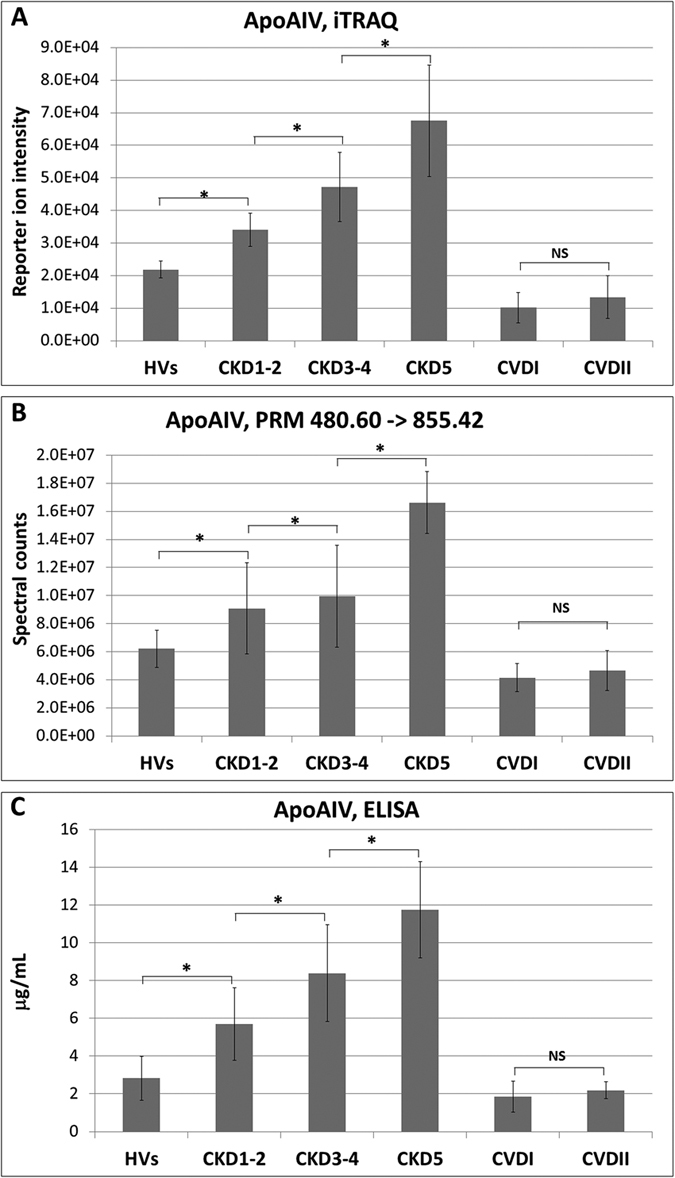
(**A**) Relative abundance of apoAIV in HVs, CKD1-2, CKD3-4, CKD5, CVDI and CVDII groups based on reporter ion intensities. (**B**) Relative abundance of apoAIV in experimental groups based on PRM analysis showing transition 480.60 m/z to 855.42 m/z. (**C**) ELISA measurements of apoAIV. Charts show mean and SD for all analyzed plasma samples. Anova and Student’s t-tests were completed and statistical significance is indicated (*P < 0.05, NS-non-significant).

In the next step, quantitative differences between all of the analyzed groups of patients were considered. The first set of differentially expressed proteins consisted of those that were inversely regulated in CVD and CKD, including apoAIV, adiponectin, and von Willebrand factor (VWF). The accumulation of apoAIV was 1.55-, 2.16-, and 3.09-fold higher in CKD1–2, CKD3–4, and CKD5 patients compared with HVs, respectively (Table 1, Fig. 5A). The up-regulation of this protein in CKD patients was correlated with CKD progression (r = −0.71). Compared with HVs, the relative abundance of apoAIV was decreased in the plasma of CVD patients together with decreased eGFR levels (r = 0.64). VWF showed the largest difference in abundance. VWF was undetectable in HVs and was higher in the plasma of CKD compared with CVD patients. Levels of this protein were 2.24- and 2.34-fold higher in the plasma of CKD1-2 patients and 3.37- and 3.52-fold higher in the plasma of CKD5 patients compared with CVDI and CVDII patients, respectively.

The second set of differentially expressed proteins consisted of those that significantly differed only between CKD patients and HVs, but not between CVD patients and HVs, and comprised apoB, apoCI, apoCII, apoCIII, apoL1, and apoH (Table 1).

The third set of altered proteins consisted of those that considerably differed between CVD patients and HVs but not between CKD patients and HVs, and comprised apoE and apoM. Deficiencies of both proteins were revealed in CVD patients. Although no significant differences were observed when comparing HVs and CKD patients, alterations in the level of apoE between CVD and CKD (especially CKD1-2 and CKD3–4) patients were evident (Table 1).

The fourth set included proteins with altered abundance in both CKD and CVD patients compared with HVs but with differences between CKD and CVD patients. Large differences, especially between CKD5 and CVD patients, were visible when comparing the accumulation of apoB, apoCI, apoCIII, apoF, apoH, and lipoprotein a (LPa). Among the 29 proteins related to lipid metabolism, 16 exhibited statistically significant differences between CKD5 and CVD patients (Table 1).

The fifth set of differentially expressed proteins consisted of apoF, apoH, haptoglobin, angiogenin, antigen CD14, and fibrinogen *α*, *β*, and *γ*. The accumulation of these proteins was increased in both CKD and CVD patients compared with HVs, but these differences were more significant in CKD patients (Table 1).

Finally, the sixth set included proteins with altered abundance between CVDI and CVDII patients. Only 9 proteins differentiated these group of patients, among them cholinesterase, angiogenin, adiponectin, serum amyloid A, and A1 and A2 proteins. The levels of cholinesterase, adiponectin, and angiogenin were up-regulated in CVDI patients compared with CVDII patients (fold changes of 2.08, 1.68, and 1.66; P = 0.01, 0.02, 0.02). The relative abundance of serum amyloid A, A1, and A2 was down-regulated in the CVDI group compared with the CVDII group (fold changes of 0.16, 0.28, and 0.21; P = 0.001, 0.03, 0.03).

To confirm the up- or down-regulation of the proteins identified in the iTRAQ analysis, PRM experiments were performed. Using the PRM approach, we successfully validated the differential accumulation of the following proteins: apoAIV, Fb *γ*, apoAI and apoB100. Two reaction monitoring transitions were determined for each protein on the basis of m/z values and retention times obtained with the PD software. The determination of the area under the curve of fragment ions confirmed the results obtained from the iTRAQ labeling. The fold changes derived from the PRM approach are presented in Supplementary material 1B. Result for PRM analysis for apoAIV is presented on Fig. 5B. An example LC-MS/MS PRM chromatogram and spectrum are presented in Supplementary Material 1C. The altered abundance of apoAIV was also confirmed by the ELISA method, and this result is presented on Fig. 5C.

### Influence of CKD progression on abundance of differential proteins associated with lipid metabolism

In the subsequent step we analyzed influence of CKD progression on observed changes in accumulation of proteins related to lipid transport and metabolism. Correlation coefficients were calculated between eGFR measures of all analyzed patients and reporter ion intensities determined for 29 differentially expressed proteins. The abundance of apoF, apoH, angiogenin, and antigen CD14 was negatively correlated with eGFR (r = −0.67, −0.51, −0.56, −0.52 for apoF, apoH, angiogenin, and antigen CD14, respectively). Weak correlations between eGFR and apoCII (r = −0.42), fibrinogen *β* (Fb *β*) (r = −0.48), and fibrinogen *γ* (r = −0.41) were also determined.

### Relationship between CVD progression and abundance of differential proteins associated with lipid metabolism

Because most of the apolipoproteins identified as differentially expressed build high-density or low-density lipoproteins, correlation analysis between the relative abundance of these plasma apolipoproteins and the HDL or LDL fractions was performed for all of the analyzed individuals. When we analyzed CKD patients, CVD patients, and HVs, only a weak correlation between apoAI, apoB100, and LDL (r = 0.199 and r = 0.451, P < 0.05) was observed. In the next step, correlation analysis was performed for CVDI patients, CVDII patients, and HVs. A statistically significant positive correlation between apoAIV and HDL (r = 0.57) and a negative correlation between apoAIV and LDL (r = −0.44) were obtained for the CVD patients and HVs. Additionally, apoAI was found to be related to the level of HDL (r = 0.41, P < 0.05), and a weak correlation between apoCI and HDL was observed (r = 0.22, P < 0.05). Finally, a correlation analysis was performed for CKD patients and HVs. The relative abundances of apoAIV and adiponectin were negatively correlated with HDL (r = −0.59 and −0.51, P < 0.05), whereas the abundance of apoAI was positively correlated with HDL (r = 0.51, P < 0.05).

## Discussion

iTRAQ based proteomic quantification is one of the most effective method for analyzing changes in plasma proteomes of diseased cells and tissues^12^. However several instruments and software for analysis of iTRAQ labeled samples have been developed in recent years. In this study we compared results from iTRAQ analyses using two different platforms: ESI-nanoLC-MS/MS and MALDI-nanoLC-MS/MS and three software solutions (Fig. 1). Finally only proteins identified in both platforms and all software were considered as significant what constituted additional validation of obtained results. Differences were revealed in number of total identified proteins (Fig. 2A). These differences may be explained by use of different search engines, PD engaged SEQUEST, whereas for PS MASCOT and for MQ Andromeda engines were in use. However, variation in identification of differentially expressed proteins between used software was very small and ranged between 5.7% and 14.3% (Fig. 2b). Additionally, the obtained results revealed a high level of run-to-run and sample-to-sample reproducibility, what was evaluated by scatter plotting and correlation coefficient analyses (Fig. 3).

CVD is the major cause of mortality in patients with CKD. All patients with CKD have an increased risk of death from cardiac events, but this phenomenon is especially evident in ESRD patients receiving renal replacement therapy^13^. However, patients with CKD differ significantly from CVD individuals with normal kidney function because of associations between blood cholesterol and vascular risk that are often atypical in CKD. Characteristic lipid profiles were also observed in our study; specifically, CKD5 patients exhibited the lowest total cholesterol and LDL levels compared with other patients and even HVs, despite the high incidence (59%) of myocardial infarction and stroke (Table 2). In patients with moderate or severe CKD, higher values of total cholesterol were observed. Similar atypical results were observed for triglyceride levels. Our results revealed that among 130 differentially expressed proteins, 29 were involved in lipid metabolism and atherosclerotic plaque formation, including diverse classes of apolipoproteins, structural components of lipoprotein particles, HDL, LDL, and VLDL. Also results from the bioinformatics analysis revealed that the most overrepresented proteins were proteins related to cardiovascular disease and atherosclerosis. For this reason in this paper we concentrate only on this set of differential proteins which are related to abnormalities in lipid transport, metabolism and atherosclerotic plaque formation. Identified apolipoproteins are anti-atherogenic (apoAI, apoAII, apoAIV, apoCI, apoCII, and apoCIII) or atherogenic (apoB100, apoF) factors; however, our results showed that this simple division did not explain the higher incidence of atherosclerosis and cardiac events in CKD patients. In our earlier studies we performed 2DE/MS analyses of plasma samples of patients with diagnosed CKD and CVD as well as in HVs and we have shown that CKD-related atherosclerosis (CKD-A) is more accelerated by inflammatory processes compared with nonrenal classical CVD^14^. We have also demonstrated that apoAIV displays an anti-atherogenic effect only in the case of classical CVD and is not efficient in CKD-A. In that work due to limited resolution of 2DE, apart from apoAIV, two additional proteins related to lipid metabolism (apoB and apoAI) were identified as differentially expressed^15^. In current, high throughput study, we discovered that the majority of apolipoprotein classes and other proteins involved in lipid metabolism revealed a completely distinct profile in CKD-A compared with CVD. Moreover we confirmed differential accumulation for proteins presented in previous study using completely different methods what constitutes additional evaluation of obtained earlier results.

ApoAIV and adiponectin show anti-atherogenic and antioxidative properties and are negatively correlated with cardiovascular disorders^16^,^17^. Adiponectin probably protects against cardiac remodeling by attenuating myocardial hypertrophy^18^; furthermore, it also has protective effects against different vascular disorders, such as endothelial dysfunction and hypertension, and inhibits platelet aggregation and inflammation (reviewed by ref. 19). Therefore, the decrease in adiponectin in the CVD group in our study was expected; however, the increase in this protein in CKD patients was surprising, given its roles in endothelial dysfunction and atherosclerosis development. ApoAIV showed similar alterations, which have previously been demonstrated^14^,^20^. Here, we additionally showed that apoAIV was positively correlated with HDL levels when comparing HVs and CVD patients; however, both proteins showed a reverse, negative correlation with HDL in matched HVs and all CKD patients. Furthermore, the negative correlation between apoAIV and eGFR in CKD patients and HVs and the positive correlation between apoAIV and eGFR in CVD patients and HVs supported the notion that the association between lipid abnormalities and risk of CVD is much less clear in this disease.

ApoE and apoM are other components of lipoproteins with recognized atheroprotective functions^21^,^22^, and, accordingly, deficiency of both proteins has been revealed in CVD patients. However neither apoE nor apoM were altered in CKD patients, even in CKD5 patients with the most advanced atherosclerosis. In contrast, apoCI, apoCII, and apoCIII, which are primarily associated with anti-atherogenic HDL particles, were differentiated in CKD patients and HVs. Also, apoB, the major constituent of atherogenic lipoproteins (VLDL, IDL, and LDL), was clearly increased in CKD5 patients compared with both CVD groups. Observed in this study the absence of a correlation between apoB and LDL may suggest that in patients with CKD, LDL is more atherogenic, due to its enhanced abundance of apoB, even when its level is not elevated. In addition to apoB, LPa is also a strong risk factor for CVD and atherosclerosis^23^. Therefore, the high level of LPa in CVD was expected, but differences between CKD and CVD were clear and were not predictable on the basis of cholesterol level.

Among the 29 proteins related to lipid metabolism, 16 revealed statistically significant differences between the groups with the most advanced atherosclerosis symptoms, i.e., the CKD5 and CVD groups. Large differences in apoB, apoCI, apoCIII, apoF, apoH, and LPa between CKD5 and CVD patients were found, underlining the fact that atherosclerosis in patients with CKD is a different process than that in patients with classical CVD.

The altered accumulation of some of the differentially expressed proteins may be explained by kidney function decline. The abundance levels of apoF, apoH, angiogenin, antigen CD14, apoCII, Fb *β*, and Fb *γ* were correlated to eGFR. These results suggest that some symptoms of lipid metabolism disorders may be affected by the progression of kidney disorder. Our previous research has shown that CVD patients exhibit an alteration in the abundance of some proteins related to nephropathy despite the lack of clinical symptoms of renal dysfunction^14^. Therefore, in this study we divided the CVD patients into two subgroups according to eGFR to determine whether an altered abundance of proteins associated with lipid metabolism is connected with kidney disorders. The fact that only nine proteins were differentially expressed between CVDI and CVDII patients, and among them only angiogenin was associated with eGFR highlights the notion that the majority of lipid metabolism proteins are altered due to atherosclerosis, not kidney dysfunction. Nonetheless, these alterations were completely distinct between CKD-A and classical CVD.

A key mechanism by which HDL exerts its anti-atherogenic effect is reverse cholesterol transport, which removes excess cholesterol from peripheral cells and subsequently transport it to the liver for catabolism^24^. A reduction in the HDL concentration may have been due to decreased apoAI and apoAII levels as a result of proteinuria; however, our results showed no correlation between apoAI or apoAII and HDL when the CVD and CKD groups were analyzed. When these groups were compared separately with HVs, only an association with apoAI was obtained, suggesting the existence of an additional mechanism related to HDL deficiency. Recently, the proper function of HDL has been suggested to be more important than HDL levels in the development of atherosclerosis^25^,^26^. HDL oxidation and sphingosine-1-phosphate content of the HDL particles have been demonstrated to be main causes of HDL dysfunction^27^,^28^. Oxidation causes a cross-linking of apoAI protein and a subsequent conformational change of HDL particles. This novel finding is in line with the fact that CKD is characterized by systemic inflammation and oxidative stress. There is substantial evidence that paraoxonase 1 (PON1) may be implicated in these processes. PON1 is transported via HDL binding to apoA1 as an athero-protective protein with anti-oxidative properties. PON1 displays a protective effect against lipoprotein macrophages and erythrocyte oxidation^29^,^30^. Recent studies have shown that oxidative stress alters PON1 and thus apoA1 in HDL that becomes dysfunctional^31^,^32^. Some studies have also shown that HDL quantity and function are markedly reduced in patients on dialysis, leading to increased oxidative stress, inflammation, and consequent progression of CVD^25^. Our study showed that the PON1 concentration was lowest in the CKD3-4 and CKD5 groups (Table 1), which suggests that dysfunctional HDL may also exist in patients who had not undergone dialysis.

In conclusion, performed in this study iTRAQ-based proteomic analysis reveals abnormalities in apolipoproteins and proteins involved in lipid metabolism and atherosclerotic plaque formation in CKD-A. The obtained results strongly indicated that atherosclerosis in patients with CKD is a different process than that in patients with classical CVD, as well as that alterations in eGFR do not reflect the observed changes in protein accumulation. The finding that apoB increased with CKD progression and the absence of a correlation between apoB and LDL suggested that even if LDL concentration is normal, its nature may be more atherogenic in CKD compared to CVD. Moreover, the inverse relationship between the level of HDL particles and their anti-atherogenic constituents suggested that dyslipidemia in dialyzed and non-dialyzed CKD patients is characterized by more qualitative than quantitative abnormalities. Patients with CKD are often excluded from large trials associated with clinical cardiovascular outcomes, and no studies have demonstrated alterations between CKD and CVD. Our unique results demonstrated that only a direct analysis of both conditions may provide information on differences in the molecular mechanism of CKD-A.

## Methods

### Subjects and samples

The study protocol conformed to the ethical guidelines of the World Medical Association Declaration of Helsinki. Before the project commenced, appropriate approval was obtained from the Bioethical Commission of the Karol Marcinkowski University of Medical Sciences, Poznan, Poland (no. 14/07 04.01.2007). All of the participating individuals provided signed informed consent for treatment and study. The characteristics of the studied population are presented in Table 2. All of studied subjects were non-albuminuric and non-diabetic. The study involved 180 individuals who were divided into six equal groups. The patients were matched for age, gender and disease. The majority were patients with CKD (90 individuals) who were treated in the Department of Nephrology, Transplantology and Internal Medicine at Poznan University of Medical Sciences. Based on the Kidney Disease: Improving Global Outcomes^1^ and the National Institute for Health and Care Excellence^2^ guidelines, the examined CKD patients were divided into three groups according to their estimated GFR (eGFR), which was calculated by the formula developed by Levey *et al*. 1999^33^. The first group, CKD1–2, contained patients in the initial stages of CKD with eGFR = 77.04 +/− 22.9 mL/min/1.73 m^2^ (mean +/− SD). The second group, CKD3–4, included pre-dialyzed patients with eGFR = 19.1 +/− 8.0 mL/min/1.73 m^2^. The third group, CKD5, consisted of ESRD patients with eGFR = 5.75 +/− 7.1 mL/min/1.73 m^2^ who were hemodialyzed for 39.6 +/− 9.5 months, three times per week. The CKD patients varied in the progression of atherosclerosis and the percentage of cardiovascular events. The CKD1-2 group showed early symptoms of hypertension or ischemic heart disease. In patients with more advanced stages of CKD, mild and severe cardiovascular disease symptoms were observed. Fifty-nine percent of CKD5 patients had a history of myocardial infarction or stroke. The underlying renal diseases were hypertensive nephropathy (n = 33), chronic glomerulonephritis (n = 21), chronic interstitial nephritis (n = 21), polycystic kidney disease (n = 3), and other/unknown (n = 12). The fourth and fifth groups (CVDI and CVDII) included 60 patients with a history and symptoms of atherosclerotic occlusive disease who were admitted for angiography to the Department of Internal Medicine, Division of Cardiac Intensive Care in Poznan University of Medical Sciences. All of the CVD patients had stenosis in at least one artery causing lumen reduction of at least 50%. Sixty-eight percent of the patients had a history of myocardial infarction or stroke. The CVD patients were divided into two subgroups. The first subgroup, CVDI, included individuals with eGFR below 90 mL/min/1.73 m^2^ (mean eGFR was 78.58 mL/min/1.73 m^2^). The second subgroup, CVDII, included individuals with normal eGFR, i.e., 90 mL/min/1.73 m^2^ or higher (mean eGFR was 106.7 mL/min/1.73 m2). A sixth group, which served as a control group (HV), contained 30 healthy volunteers with a mean eGFR of 123.6 mL/min/1.73 m^2^. Individuals with diabetes mellitus, acute inflammatory processes, and malignant tumors either at the time of study or within the previous 10 years were excluded from the study. All of the studied subjects were diagnosed with atherosclerosis on the basis of their medical history (history of myocardial infarction and/or ischemic stroke), systolic and diastolic blood pressure values, lipid metabolism parameters, and carotid intima media thickness (CIMT). Although, patients participating in this study received the recommended medications, such as angiotensin-converting enzyme inhibitors (ACEI), non-steroidal anti-inflammatory drugs (NSAID), beta-blockers and statins, that were related to their hypertension, history of cardiovascular disease, and lipid profile disturbances, not all of the them received all of these medications together, and during the same period of time. Adjustment of the studied groups in this respect appears to be virtually impossible, primarily due to the nature of the underlying diseases and their different duration time. However, to achieve the goal of this study, in order to minimize the differences between the groups in this regard, we put particular attention on these of the drugs that affect lipid metabolism. For this purpose, patients who received drugs other than statins, like for example fibrates (due to the small number of patients; n = 7) were excluded from the study. In addition, patients who received the highest doses of statins were also excluded. Moreover, we included to the study only these patients with defined time period of statins administration (25 +/− 10 months), to minimize the influence of this factor on the results. However the observed differences between the groups in the treatment, which we could not avoided, did not have a statistically significant impact on the obtained results. Similar conclusions about this topic were presented in our previous article^14^. Therefore, more detailed information about medications has been omitted from this study. Peripheral blood was collected in a closed monovette system containing EDTA and was immediately centrifuged at 1,000 g for 15 min. The obtained supernatants were then centrifuged at 16,000 g for 15 min at 4 °C and frozen at 80 °C.

### Protein digestion and iTRAQ labeling

The protein concentrations of all plasma samples were determined using the BCA (Thermo Fisher Scientific, USA) method. Then, 25 *μ*g of plasma proteins derived from three individuals from the same experimental group were pooled into one sample. Protein digestion before iTRAQ labeling was performed on 75-*μ*g plasma protein aliquots according to the manufacturer’s instructions (AB Sciex, USA) with the following minor modifications. To each sample containing 75 *μ*g of plasma proteins, 20 *μ*L of 100 mM triethylammoniumbicarbonate (TEAB) at pH 8.5 was added. The solution was then mixed for 5 min and sonicated in an ice-water bath for three, 1-min cycles. Proteins were reduced with 4.5 mM TCEP for 1 h at 60 °C. Then, the samples were incubated in 2 mM iodoacetamide for 30 min in the dark to block reduced cysteine residues. After alkylation, the proteins were digested with 2 *μ*g of sequencing-grade trypsin (Promega, Germany). Digestion was performed overnight at 37 °C. After digestion, 15 *μ*l of 1 M TEAB was added to the samples. For labeling, each iTRAQ reagent (AB Sciex, USA) was dissolved in 50 *μ*l of isopropanol and added to the respective peptide mixture for 120 min. In all of the iTRAQ experiments, all of the analyzed groups were labeled with the same iTRAQ tag as follows: 113 - CKD1–2, 114 - CKD3–4, 115 - CKD5, 116 - CVDI, 117 – CVDII, and 121 iTRAQ tag for healthy volunteers (HVs). The labeling reaction was quenched by the addition of 100 *μ*l of MilliQ water, and six labeled samples were then pooled into one sample according to the manufacturer’s instructions. After pooling, the samples were evaporated to 50 *μ*l by vacuum concentration to remove excess water, TEAB, and isopropanol. The labeled digest was purified and fractionated on an SCX cartridge system (AB Sciex, USA) in an off-line manner. The peptides were sequentially eluted from the column with increasing KCl concentrations. Four fractions were collected in 87.5, 175, 350, and 500 mM KCl using 350-*μ*l aliquots of KCl in 10 mM KH_2_PO_2_ and 25% (v/v) acetonitrile. The collected fractions were finally desalted on SPE BakerBond^TM^ C18 Cartridges (J.T. Baker, USA) and then evaporated to 50 *μ*l by vacuum concentration to remove the acetonitrile. Ten different iTRAQ experiments were performed. In each experiment, three samples from the same experimental group were pooled into one sample and labeled with one iTRAQ reagent. Thus, 18 samples derived from 6 groups were analyzed in each experiment (as four KCl fractions). Finally, 180 samples were analyzed in all ten iTRAQ experiments.

### ESI-NanoLC-MS/MS analysis

For each iTRAQ experiment, 5 *μ*l of respectively KCl fraction was injected on a RP C18 precolumn (Thermo Fisher Scientific, USA) connected to a 75-*μ*m i.d. ×25 cm RP C18 Acclaim PepMap column with 2-*μ*m particles and a 100-Å pore size (Thermo Fisher Scientific, USA) using a Dionex UltiMate 3000 RSLC nano system (Thermo Fisher Scientific, USA). Each fraction was injected in triplicate. Every 20 sample injections, the system was calibrated using Pierce LTQ ESI Positive Ion Calibration Solution (Thermo Fisher Scientific, USA). The following LC buffers were used: buffer A (0.1% (v/v) formic acid in Milli-Q water) and buffer B (0.1% formic acid in 90% acetonitrile). The peptides were eluted from the column at a constant flow rate of 300 nL/min with a linear gradient of buffer B from 5 to 65% over 208 min. At 208 min, the gradient was increased to 90% B and was held there for 10 min. Between 218 and 230 min, the gradient was returned to 5% to re-equilibrate the column for the next injection. Peptides eluted from the column were analyzed in data-dependent MS/MS mode on a Q-Exactive Orbitrap mass spectrometer (Thermo Fisher Scientific, USA). The instrument settings were as follows: the resolution was set to 70,000 for the MS scans and 17,500 for the MS/MS scans to increase the acquisition rate. The MS scan range was from 300 to 2,000 m/z. The fixed first mass was m/z 100.0. The target value was 1e6, and the maximum injection time was 100 ms. The number of microscans was 1, and the ion selection threshold was 5e4. To reduce the interference of precursor co-fragmentation with the iTRAQ quantification, the isolation window was set as 1.2 m/z.

### Maldi Off-line LC-MS/MS analysis

The samples from each of the ten iTRAQ experiments were subjected to nano-LC separation using an EASY-nLC Proxeon (Bruker Daltonics, Germany) coupled to a Proteineer fc II (Bruker Daltonics, Germany) fraction collector as previously described^34^. For each iTRAQ experiment, 5 *μ*l of respectively KCl fraction was injected in triplicate. In total, 384 fractions were collected. The UltrafleXtreme MALDI-TOF/TOF (Bruker Daltonics, Germany) instrument was operated in the positive ion mode and controlled by the Compass for Flex software, version 1.3 (FlexControl 3.3, FlexAnalysis 3.3, Bruker Daltonics, Germany). Five thousand laser shots were accumulated per spectrum in the MS and MS/MS modes. The spectrometric analysis was performed in automatic data-dependent mode. The nonredundant precursor peptides were selected for MS/MS analysis using the WARP-LC 1.3 software (Bruker Daltonics) with a signal-to-noise threshold of 12. The MS spectra were externally calibrated using the Peptide Calibration Standard mixture (Bruker Daltonics).

### Analysis of iTRAQ data

Raw files derived from ESI-LC-MS/MS were analyzed in Proteome Discoverer (PD) version 1.4.14 (Thermo Fisher Scientific) and MaxQuant (MQ)^35^,^36^ software version 1.5.1.2. For protein identification, the PD SEQUEST search engine was used to search MS/MS spectra against the UniProt Complete Human Proteome Set (123,619 sequences) database. The Percolator software integrated in the PD was used to evaluate the database search results. To evaluate the quality of the performed runs, the number of peptide spectrum matches (PSMs) and the number of identified proteins were calculated. The LC-MS/MS runs with the number of PSMs below 125,000 and the number of identified proteins below 450 (with 1% FDR) were excluded from further analysis. For protein identification in MQ, the database search engine Andromeda was used to search MS/MS spectra against the same UniProt database. The obtained MALDI data were analyzed using the ProteinScape (PS) (Bruker Daltonics) database software, and searched with MASCOT 2.3 (Matrix Science, London, UK) against the UniProt database. The false discovery rate (FDR) for peptide identification in PD, MQ, and PS was set at 1%. The parameters for database searching were as follows: iTRAQ 8plex (peptide-labeled) modification and tolerance levels of 10 ppm for MS, 0.05 Da for MS/MS (ESI-LC-MS/MS), 0.3 Da for MS, and 0.5 Da for MS/MS (MALDI-LC-MS/MS). Carbamidomethylation of cysteines was set as a fixed modification, and oxidation of methionine was allowed as a variable modification. Trypsin was used as the enzyme, and two missed cleavages were allowed. All identifications based on only one unique peptide were eliminated. The relative peptide abundance was measured using the iTRAQ reporter ion peak area ratios. The following ratios were calculated: 113/114, 113/115, 113/116, 113/117, 113/121, 114/115, 114/116, 114/117, 114/121, 115/116, 115/117, 115/121, 116/117, 116/121, and 117/121. Normalization is carried out by calculation of median values for all peptides for each group (each label) and then intensities are recalculated according to an average of medians of all labels. Additionally, the correction of reporter ion intensities based on isotopic impurity was done as well as filtration of other interfering precursor ions (PIF). The data from MQ were evaluated, and statistical analysis was performed using the Perseus software (version 1.4.1.3, Max Planck Institute of Biochemistry, Martinsried). MQ data were filtered for reverse identifications (false-positives), contaminants, and data that were ‘only identified by site’. The mean values +/− SD were calculated from the reporter peak area ratios of all labeled peptides for a given protein. Proteins with a fold change of at least 1.5 identified by PD, MQ/Perseus, and PS were considered to be differentially expressed and were then statistically analyzed.

### Assessment of variability/reproducibility and statistical analysis

The reproducibility of technical and biological replicates was assessed by scatter plotting and correlation coefficient determination based on reporter ion signals. The percentage overlap in protein identification between both technical/injection and biological replicates was calculated. Coefficient of variation (CV) values were the primary parameters used to validate the data. Proteins with variability in reporter ion signals above 30% were excluded from the analysis as described earlier^37^. Additionally, during statistical analyses all proteins were filtered and only hits present in at least 75% of samples were retained. Positively evaluated reporter ion intensities derived from all samples and from all 10 experiments were considered in the statistical analysis. For multiple comparisons, one-way analysis of variance (ANOVA) with Bonferroni correction for multiple testing was performed. For comparisons between two groups, t-tests were performed. P values < 0.05 were considered to be statistically significant. For the obtained results, regression and correlation analyses were also performed. Correlations between variables were assessed on the basis of Pearson (Perseus) and Spearman (Statistica) coefficients, and P values < 0.05 were considered significant. Multivariate analyses were carried out by untargeted principal component analysis (PCA). All of the statistical analyses were performed using Statistica v. 10.0 software (StatSoft, Inc., www.statsoft.com) and Perseus 1.4.1.3 which is freely available from the MaxQuant website.

### Validation of differentially expressed proteins

Parallel reaction monitoring analyses were performed using a Q-Exactive mass spectrometer. PD software was used to identify proteins and to produce a list of identified peptides containing m/z values and retention times for subsequent PRM analysis. Four proteins that were identified as differentially expressed were validated using the PRM approach. Two reaction transitions were empirically determined for each protein. The transitions were calculated based on the analysis of PSM counts in the iTRAQ experiments. The plasma samples derived from seven individuals from each experimental group were analyzed. Plasma proteins (10 *μ*g) were digested without labeling as described earlier^15^. For each run, 3 *μ*g of the digest was injected and separated as described in the previous section. Peptides eluting from the column were analyzed by a Q-Exactive Orbitrap mass spectrometer. The acquisition method involved the combination of scan events corresponding to a full MS scan and a PRM event with inclusion list. The instrument settings for the MS scans were as follows: resolution, 17,500; scan range, 150 to 2000 m/z; target value, 3e6; maximum injection time, 200 ms; and number of microscans, 1. The PRM method entailed an orbitrap resolution of 17,500, a target AGC value of 5e4, and maximum fill times of 600 ms. The precursor ion of each targeted peptide was isolated using a 1.6-m/z unit window (a list of target peptides, target precursor ions, and selected fragment ions is provided in Supplementary data 1D). Data analysis was performed using Xcalibur (version 3.0, Thermo Fisher Scientific, USA). The peaks of fragment ions were extracted with 10 ppm mass tolerance and Gaussian smoothing. The determination of the area under the curve of selected fragment ions was performed manually.

An ELISA was used to validation of apoAIV abundance. The plasma protein level was measured using a commercially available sandwich colorimetric ELISA kit (Elabscience, China). Assay was prepared according to the manufacturer’ instructions. The O.D. absorbance was read at 450 nm with an Infinite 200 PRO multimode reader (Tecan, Switzerland).

### Functional annotation of dysregulated proteins in plasma samples

Only the proteins that were quantified as unique and non-redundant were retained in the subsequent analysis. Proteins were considered to be differentially expressed if the difference was statistically significant (P<0.05) and the fold change was >1.5 or <0.66. The dysregulated proteins were subjected to analysis with the DAVID (the Database for Annotation, Visualization, and Integrated Discovery) (http://david.abcc.ncifcrf.gov/) tool for identification of enriched disease categories^38^. P values and Benjamini corrected P values <0.05 were considered to be significant. Disease analysis in DAVID was performed based on the GENETIC ASSOCIATION DB DISEASE database.

## Additional Information

**How to cite this article**: Luczak, M. *et al*. iTRAQ-based proteomic analysis of plasma reveals abnormalities in lipid metabolism proteins in chronic kidney disease-related atherosclerosis. *Sci. Rep.* **6**, 32511; doi: 10.1038/srep32511 (2016).

## Supplementary Material

Supplementary Information

## Figures and Tables

**Table 1:** Comparison of the abundance of proteins differentially expressed in HVs and CKD/CVD patients.

Protein names	ID	ANOVA p	CKD1-2/HV	CKD3-4/HV	CKD5/HV	CVDI/HV	CVDII/HV
Adiponectin	Q15848	0.00316	1.69*	1.34	2.05*	0.65*	0.39*
Angiogenin	P03950	2.07E-15	8.56**	13.40***	33.90***	6.39*	3.85*
Angiotensinogen	P01019	1.90E-06	1.06	0.65***	0.86	0.46**	0.45***
Antigen CD14	P08571	0.00033	2.03*	2.85**	3.68***	1.51*	1.59*
Apo A-I	P02647	1.03E-06	1.14	0.53**	0.49*	0.31*	0.36***
Apo A-II	P02652	0.00583	1.01	0.54*	0.54*	0.53**	0.58**
Apo A-IV	P06727	3.79E-31	1.55*	2.16***	3.09***	0.46***	0.61***
Apo B-100	P04114	0.00167	1.45***	1.55*	1.68*	1.11	1
Apo C-I	P02654	0.01084	1.39*	1.06	1.59*	0.87	0.73
Apo C-II	P02655	0.04411	1.46*	1.59***	1.40**	1.44	1.46**
Apo C-III	P02656	0.00099	1.33	1.59**	1.62***	1.02	1.04
Apo E	P02649	5.25E-05	1.27*	0.88	1.12	0.41*	0.49*
Apo F	Q13790	0.00938	1.56*	2.07***	1.79**	1.55*	1.58*
Apo H	P02749	3.10E-07	1.21*	1.54***	1.64***	1.26*	1.15
Apo L1	O14791	0.04986	1.50*	1.03	0.98	1.09	0.97
Apo M	O95445	0.00917	0.89*	0.78**	0.83*	0.50**	0.55**
Cholinesterase	P06276	0.00152	1.85*	0.81	0.66*	0.82	0.39*
Fibrinogen *α*	P02671	1.65E-13	1.50***	1.56***	1.71***	1.55*	1.52**
Fibrinogen *β*	P02675	1.43E-12	1.73***	2.01***	2.06***	1.50*	1.65***
Fibrinogen *γ*	P02679	1.07E-10	1.65***	1.67***	1.91***	1.57*	1.57*
Haptoglobin	P00738	0.00014	1.65*	2.08***	1.60*	1.85***	2.19***
Lipoprotein, Lp(A)	Q1HP67	0.00015	2.44*	3.73**	2.25*	4.99**	6.83***
Serum amyloid A	D3DQX7	1.98E-05	0.49*	35.57*	3.47*	3.65*	22.86**
Serum amyloid A-1	P0DJI8	0.00097	1.03	15.02*	2.65*	3.98*	13.90*
Serum amyloid A-2	P0DJI9	0.00299	absent in HV	and CKD1-2	0.04*	0.19*	0.92
Serum amyloid A-4	P35542	0.0002	1.56*	1.66**	1.98**	1.84*	2.13***
Serum amyloid P	P02743	0.02349	1.10*	1.88**	1.84*	2.22*	2.12*
Serum paraoxonase 1	P27169	1.86E-07	1.12*	0.33***	0.33***	0.50*	0.52**
von Willebrand factor	L8E853	1.21E-05	absent in HV	1.35	1.51**	0.40*	0.40*

Fold changes were calculated against the HV group; only in the case of the two proteins fold changes were calculated against the CKD1-2 (von Willebrand factor) or CKD3-4 (serum amyloid A-2) experimental group. *t-test P value < 0.05, **t-test P value < 0.005, ***t-test P value < 0.0005. Differences identified as significant (P < 0.05 and fold change >1.5 or <0.66) are in bold.

**Table 2:** Demographic data and clinical characteristics of the study population (n = 180).

	HV	CKD1-2	CKD3-4	CKD5	CVDI	CVDII	*p*
Age [years]	60.5 +/− 10.1	61.5 +/− 9.1	59.5 +/− 6.3	60.1 +/− 10.1	63.22 +/− 11.9	62.6 +/− 9.1	NS
Sex [males/females]	17/13	17/13	17/13	17/13	17/13	17/13	NS
BMI [kg/m2]	24.9 +/− 2.8	28.3 +/− 1.2	25.7 +/− 3.4	28.9 +/− 3.2	27.73 +/− 3.2	28.1 +/− 4.1	0.00
eGFR [ml/min/1.73 m2]	123.6 +/− 17	77.0 +/− 22	19.1 +/− 8	5.7 +/− 7	78.5 +/− 12	106.7 +/− 16	0.00
Arterial hypertension	0%	100%	100%	100%	100%	100%	0.00
History of MI/stroke	0%	15%	21%	59%	68%	68%	0.00
Statin treatment	0%	61%	49%	68%	92%	92%	0.00
ACEI treatment	0%	84%	48%	60%	76%	76%	0.00
Total cholesterol [mg/dL]	185.1 +/− 29	229 +/− 51	184.3 +/− 28	179.5 +/− 38	189.9 +/− 39	186.7 +/− 35	0.03
HDL cholesterol [mg/dL	70.1 +/− 5.1	55.1 +/− 10.1	58.4 +/− 7.6	45.1 +/− 23.0	40.5 +/− 11.5	51.2 +/− 11.7	0.00
LDL cholesterol [mg/dL]	91.9 +/− 29.1	169.6 +/− 41.2	120.0 +/− 17.1	103.2 +/− 40.0	115.9 +/− 32.8	107.0 +/− 30.6	0.00
Triglycerides [mg/dL]	121.2 +/− 34	170.4 +/− 68.1	117.2 +/− 21.1	133.3 +/− 50.8	134.4 +/− 63.3	138.4 +/− 75.5	0.04
CIMT [mm]	0.45 +/− 0.2	0.71 +/− 0.2	0.83 +/− 0.2	0.92 +/− 0.4	0.72 +/− 0.3	0.72 +/− 0.3	0.00
Urea [mg/dL]	27.6 +/− 8.7	31.4 +/− 15.8	92.8 +/− 41.2	125.5 +/− 32.8	38.2 +/− 12.2	29.4 +/− 11.9	0.00
Uric acid [mg/dL]	4.1 +/− 1.6	5.9 +/− 2.2	8.2 +/− 3.4	9.2 +/− 7.2	6.4 +/− 3.1	4.6 +/− 2.8	0.00
Cystatin C [mg/L]	0.5 +/− 0.1	0.99 +/− 0.3	1.92 +/− 0.5	3.0 +/− 0.6	1.0 +/− 0.3	0.77 +/− 0.1	0.00
HGB [g/dL]	14.1 +/− 0.9	13.3 +/− 3.1	11.5 +/− 1.2	11.3 +/− 1.1	13.8 +/− 1.2	14.0 +/− 0.5	0.00
RBC [10^12^]	4.7 +/− 0.4	4.6 +/− 0.4	3.9 +/− 0.6	3.6 +/− 0.5	4.4 +/− 0.6	4.6 +/− 0.5	0.00
Glucose [mg/dL]	75.1 +/− 9.2	82.2 +/− 8.9	81.9 +/− 9.12	81.8 +/− 9.5	79.1 +/− 7.2	81.5 +/− 6.2	NS
hsCRP [mg/L]	1.0 +/− 0.1	1.6 +/− 0.3	9.1 +/− 2.1	12.3 +/− 18.0	5.8 +/− 1.0	2.1 +/− 0.1	0.00
Total protein [g/dL]	7.2 +/− 0.4	6.6 +/− 1.7	5.5 +/− 1.4	6.6 +/− 0.6	7.1 +/− 0.4	7.1 +/− 0.2	0.00
Albumins [g/dL]	4.2 +/− 0.5	4.0 +/− 0.9	4.1 +/− 1.7	3.8 +/− 0.6	4.2 +/− 0.5	4.1 +/− 0.2	0.00
Ca total [mg/dL]	8.6 +/− 0.2	7.1 +/− 2.7	8.0 +/− 0.4	9.6 +/− 2.2	8.6 +/− 0.2	8.4 +/− 0.2	NS
PO_4_^3−^ [mg/dL]	3.4 +/− 0.7	3.6 +/− 1.8	3.7 +/− 1.5	7.0 +/− 3.5	3.7 +/− 0.8	3.0 +/− 4.2	0.00
iPTH [pg/mL]	38.1 +/− 6.5	89.4 +/− 23.1	198 +/− 135.2	320 +/− 202	38.2 +/− 5.6	39.4 +/− 7.3	0.00

Mean value +/− SD. P < 0.05 was considered to be statistically significant, NS - indicates no significant differences between the studied groups. eGFR - estimated glomerular filtration rate, MI - myocardial infarction, BMI - body mass index, ACEI - angiotensin-converting enzyme inhibitors, HDL cholesterol – high-density lipoprotein cholesterol, LDL cholesterol – low-density lipoprotein cholesterol, CIMT - carotid intima media thickness, HGB - hemoglobin, RBC - red blood cells, hsCRP - high-sensitivity C-reactive protein, iPTH - intact parathormon, PO_4_^3−^ - phosphates, Ca total- total calcium.
